# Supplementary material for: Are carbon emissions trading and green financial instruments synergistic? -Comprehensive quantitative research based on content analysis
Source: PLoS One. 2024 Mar 7;19(3):e0298601. doi: 10.1371/journal.pone.0298601 (PMC10919601; doi:10.1371/journal.pone.0298601)
Supplement: S1 Data — (ZIP) [file pone.0298601.s001.zip › Data/地级市Z_F工作报告.zip!/║╙─╧/╓▄┐┌╩╨/└┤╫╘╣1⁄2╓┌║┼í╛┬φ┐╦╩2╛▌═°í┐_2009.docx]

各位代表：

　　现在，我代表市人民政府，向大会报告政府工作，请予审议，并请各位政协委员和其他列席人士提出意见。

　　一、２００８年工作回顾

　　２００８年是周口奋力追赶跨越的一年。面对复杂多变的形势，在省委、省政府和市委的正确领导下，市人民政府以科学发展观为统领，团结带领全市人民，坚定不移地贯彻执行中央的宏观调控政策，坚持从实际出发创造性地开展工作，千方百计保增长，全力以赴保民生，较好完成了市二届人大三次会议确定的各项目标任务，经济社会保持了又好又快的发展态势。预计全市生产总值完成９８４．１亿元，增长１２．３％，其中第一产业增加值２９８．２亿元，增长５．７％；第二产业增加值４２０．９亿元，增长１４．３％；第三产业增加值２６５亿元，增长１６．２％。地方财政一般预算收入２５．５亿元，增长２９．７％，其中税收收入１７．９亿元，增长３５％；市本级一般预算收入６．３亿元，增长４１．５％，其中税收收入４．７亿元，增长５３．８％，４项财政收入指标增幅均居全省第一位。地方财政一般预算支出１２９．３亿元，增长２８．９％。城镇居民人均可支配收入１０４０６元，实际增长８．７％；农民人均纯收入３６０５元，实际增长７．５％。金融机构各项存、贷款余额分别为 ６７３．１亿元和３９５．３亿元，较年初分别增加１０５．３亿元和３５亿元。

　　一年来的主要工作和成效是：

　　——强力实施项目带动战略，经济社会发展后劲显著增强。将投资作为加快发展第一启动力，出台关于加强项目建设、扶持房地产发展、扩大信贷投放等４个文件，采取扩大招商引资、赢得银行支持、争取国家资金、激活民间资本等方式，千方百计加大投资力度，强化项目建设。美国玮伦、山西晋煤、江苏雨润和东华等国内外知名企业投资我市。组织企业参加银企洽谈活动４次，向各级金融机构推荐项目２１０个，为企业融资近６０亿元。争取到位各类资金项目８３９个，项目资金９．２４亿元。全社会固定资产投资５３１亿元，增长３２．７％。“双十五”工程、１００个重点项目基本完成年度投资计划，２×３０万千瓦机组项目、漯阜铁路电气化复线改造工程等一批重大项目开工奠基；采用ＢＴ 方式开工建设“两桥一路三馆”工程，办了多年想办而没有办成的事。

　　——认真做好“三农”工作，农业和农村经济全面发展。１５项惠农补贴全部兑现，共补贴资金１１．９亿元。粮食总产量１４１．２亿斤，占全省总产量的１３．１％，连续五年丰产增收，连续三年创历史新高。继沈丘县之后，商水县、淮阳县、项城市实现夏粮亩产超千斤，淮阳县、鹿邑县被评为全国粮食生产先进县。实施品牌战略，扶沟蔬菜被评为全国果蔬十大知名品牌。农业生产组织化程度不断提高，农民专业合作社发展到７８３家。“畜牧业发展推进年”活动成效明显，畜产品总量较上年增长２３％。太康县、沈丘县分别被命名为全国肉品生产百强县、全国奶业发展百强县，商水县、西华县、淮阳县、太康县、沈丘县被评为全国生猪养殖调出大县。大力推进林业生态市建设，我市被定为国家现代林业示范市，扶沟县、西华县、鹿邑县通过林业生态示范县验收。多方筹措资金８亿元，改造中低产田２３．４万亩，新增有效灌溉面积３５．１万亩、旱涝保收田２１．１万亩。西华县、鹿邑县被省政府授予“红旗渠精神杯”竞赛活动先进单位。农村生产生活条件明显改善。新建改建农村公路１２７７公里。解决２９万农村居民饮水安全问题，项城市被确定为国家级饮水安全改革试点市，并荣获“全国农村饮水安全示范市”称号。完成１０９个试点村５０１个垃圾池建设任务。新增沼气用户１０．６万户。大力发展劳务经济，劳务输出２７１万人次，劳务收入１６８亿元。

　　——全力扶持企业做大做强，工业经济质量和效益大幅提升。立足周口实际，加强服务扶持，工业经济在化解诸多不利因素中实现历史性跨越。规模以上工业增加值２３２．７亿元，增长２１．５％，增幅居全省第四位，较上年前移１１个位次；实现利润９８．３亿元，增长２０．１％。规模以上工业企业新增１５９家，总数达到７５２家，志元、益海、辅仁、莲花、财鑫５家企业跻身全省工业企业百强，祥龙四五酒业在新加坡成功上市。完成工业投资１８５．５亿元，增长４７．５％，增幅居全省第三位。投资１２．８亿元用于工业园区建设，入驻企业２２９家。产业集群呈现良好发展势头，省级重点产业集群达到６个，沈丘县、鹿邑县荣膺“中国县域产业集群竞争力百强”称号。企业技术研发能力进一步增强，金丹乳酸公司技术中心被认定为国家级企业技术中心，省级以上企业技术中心达到１６家。工业企业产品知名度不断提升，新增河南省优质产品１３个。

　　——不断拓宽服务业领域，第三产业发展水平明显提高。新建改建天下诚、新天地等大型商贸流通设施和网点１２处。万果园、一峰等超市逐步向规模化、集团化方向发展。提前完成“万村千乡市场工程”省定目标任务，新建农家店１００５家、配送中心１８家。文化产业长足发展，郸城县荣获“中国书法之乡”称号，淮阳县被确定为全省文化改革发展试验区。成功举办首届国际老子文化节，太昊陵—老子故里景区成为全省十大精品园区之一，我市通过中国优秀旅游城市验收，淮阳县、鹿邑县通过中国旅游强县验收。旅游知名度不断提升，接待国内外游客７６６．８万人次，增长３２．６％；旅游综合性收入３５亿元，增长３０．６％。市中心城区房地产开发投资１３．５亿元，新开工面积８９万平方米，销售面积５４万平方米。保险、信息、中介、社区服务等新兴服务业发展迅速。

　　——深入推进改革开放，经济发展更富活力。全市１５１家国有工业企业基本完成改制任务。商贸、物资流通改革取得新进展。财税金融改革成效明显。强化综合治税各项举措，有效堵塞了漏洞。金融机构不良贷款率较年初下降１７．５％，其中四大国有商业银行由４８．３％下降到５．４％。非公有制经济迅猛发展，总量占ＧＤＰ的比重达到５９％。深入开展“解放思想、改革创新、开放开明”主题教育活动，加快政府自身建设，取消行政审批项目７项。行政管理体制和事业单位制度改革稳步推进。水利、科技、教育、土地、交通、建设等领域的改革也迈出了新的步伐。对外开放规模和水平进一步提高。多次参加国家、省组织的经贸洽谈活动，签订投资项目９０个，合同资金９０．２亿元。新登记进出口企业１９２家。２家企业在境外投资办厂，向境外派出各类劳务人员７９０人。全市外贸进出口完成４．１亿美元，增长６８．４％，增幅居全省第四位；实际利用外资１．１亿美元，增长４３．４％，增幅居全省第三位。

　　——加快城镇化建设，城乡面貌进一步改善。完成周商一体化战略规划、周口市住房建设规划等部分规划，充实完善了东新区、旧城区、开发区规划。中心城市旧城区改造、开发区建设和东新区开发同步进行。旧城区完成八一路北段、六一路南段、新建路改造任务，维修七一路、中州路、汉阳路等主次干道沥青路面２．２万平方米，亮化改造交通路、界牌街、育新街中段等１０多条主次干道。新铺设天然气管道５万多米，发展用户１万户。开发区市综合办公楼、市卫生局、市审计局办公楼建成启用，市人大、市政协和部分市直单位办公条件得到改善。东新区市委党校新校区、第一实验小学建成投用，广电中心、报业大厦、安居小区等项目进展顺利。中心城区人均公共绿地达１０．６８平方米，成功实现省级园林城市创建目标。淮阳县、鹿邑县被命名为省级园林城。周商、周淮一体化进程加快。县（市）完成城镇基础设施建设投资６亿元，新增城镇道路１８５．５公里、供水管道９３．５公里、排水管道１１４公里、公共绿地１２３公顷，城镇功能日益完善。城镇化率达到２７．６％，较上年提高１．６个百分点。项城市李寨镇被评为全国文明村镇。大力推进县域经济发展，县域生产总值达到８９７．９８亿元，占全市生产总值的９１．２％。鹿邑县、沈丘县进入全省“十快县”，县域经济综合实力明显增强。

　　——重视解决事关群众切身利益问题，改善民生工作取得新成效。全市用于解决民生问题的财政支出５８．３亿元，占财政总支出的４５．１％。向全市人民承诺的十大实事全部完成。城市低收入家庭住房保障工作进展顺利，中心城区１２万平方米、县（市）８．６万平方米的经济适用住房开工建设，川汇区２２８套廉租住房建成。补贴低收入家庭廉租房租金１１１４万元，困难群体住房难问题得到缓解。深入开展产品质量和食品药品安全专项整治工作，维护广大人民群众切身利益，及时筛查救治了问题奶粉受害婴幼儿。

创建“白内障无障碍市”活动初见成效，为８１４０名白内障患者实施了免费手术。努力改善农村卫生服务条件，完成１１２１个标准化村卫生室建设。新型农村合作医疗参合农民８５４．７万人，参合率８９．３％。重视解决市区上学难和中小学大班额问题，在周口一高原校址新办初中部，“一建五扩”学校全部投入使用。认真落实农村义务教育“两免一补”政策，资助贫困生４０１．７万人次，资助金额９．６亿多元。就业和社会保障工作取得实效。城镇新增就业人员７．９万人，下岗失业人员再就业４．１万人，动态消除零就业家庭，城镇登记失业率控制在３．９％以内。积极推进城镇居民医疗保险试点工作，４２．４万城镇居民参加医疗保险。企业养老保险、医疗保险、失业保险覆盖面不断扩大，参保人数分别达到３３．３万人、３９．８万人和３８．６万人。养老金在标准提高的情况下，做到了按时足额发放。清理拖欠农民工工资１０３１．９万元，维护了进城务工人员合法权益。新建、改扩建５４所农村敬老院，农村五保集中供养率达到４０％。共为城乡低保对象发放低保资金３．１亿元。慈善事业健康发展，募集资金１８９０万元。家电下乡销售数量和补贴农户资金均居全省第一位。认真做好艾滋病致孤致贫人员救助和艾滋病困难家庭帮扶工作。向城市低保群众发放价格补贴２００万元。扶贫开发成效显著，全市农村又有１４万人脱贫。同时，积极支援四川灾区抗震救灾，累计捐款２２００多万元、捐赠衣被９３９３件，先后派出４支队伍３００多名救援人员赶赴第一线开展抢险救灾和对口援建，援建安置房工作的“周口经验”被中央电视台和河南电视台多次报道。

　　——积极实施科教兴市和可持续发展战略，各项社会事业全面进步。科技创新工作得到加强，除草安全添加剂奈安产品生产工艺和配方填补了国内空白，培育出农作物新品种（系）５个，７项优秀科技成果荣获省科技进步奖；进一步强化知识产权工作，申请专利３０６件。教育事业加快发展，新建民办职业学校８９所，改造建设农村中小学校舍２４．８万平方米，完成初中项目学校３９所，扶沟县、项城市被评为全省两基工作先进县（市）。成功举办周口市第一届运动会；开展“全民健身与奥运同行”活动，我市被评为全国全民健身周活动先进单位；竞技体育再创佳绩，沈丘县运动员张志磊在北京奥运会拳击比赛中勇夺银牌。加快公共文化服务体系建设，在农村放映公益电影６万多场，居全省第一位；实施文化精品工程，《都市长虹》、《云锦人家》和《老子》３个剧目获得河南省文华大奖；３项非物质文化遗产项目入选第二批国家级非物质文化遗产名录，汝阳刘毛笔入选河南省非物质文化遗产名录。公共卫生服务体系进一步健全，覆盖城乡的１２０院前急救网络建设已经形成；４个县级医院和３６所薄弱乡镇卫生院被列入国债项目建设规划；市中心医院病房大楼投入使用、市中医院门诊病房综合楼基本建成；重大疾病防治工作扎实推进，手足口病、艾滋病和结核病防治工作成效显著。加强环境保护和生态建设，累计争取国家各类环保资金７６８１万元；完成隆达电厂脱硫工程建设和重点流域１０家深度治理企业专项整治任务；９个县市污水处理设施建成使用，商水县、淮阳县、沈丘县垃圾处理场及其他６个县市和市垃圾处理填埋场投入运营；化学需氧量和二氧化硫排放量分别完成全年和“十一五”减排目标；饮用水源地取水水质平均达标率１００％；市区空气质量优良天数３４０天。土地“三项整治”新增耕地３．９万亩。稳定低生育水平，人口出生率１０．２７％。，低于省定目标１．８９个千分点。安全生产形势逐步好转，死亡人数下降６．９％；郸城县荣获全国安全生产宣传先进县。审计、监察、统计、物价、气象、人防、档案、史志、广播电视、外事侨务、爱国卫生等各项社会事业也取得了新的成绩。

　　——加强社会主义精神文明和民主法制建设，社会大局保持稳定。以创建文明城市为契机，开展不同形式的文明创建实践教育活动，城乡文明程度不断提高。加强文化市场管理，推进新闻出版业健康发展，“扫黄打非”斗争取得新成效。顺利完成第六届村委会换届选举工作。“五五”普法迈上新台阶。自觉接受人大法律监督、工作监督和政协民主监督，共办理人大代表建议８７件和政协委员提案３６５件。密切联系工会、共青团、妇联等人民团体，广泛听取社会各界意见和建议。全面贯彻实施行政许可法、行政复议法等法律法规，法治政府建设和依法行政工作扎实推进。加强政府廉政建设，反腐败斗争取得新成效。深入开展国防教育和双拥共建活动，国防后备力量建设取得新进展，优抚安置工作不断深化，商水县被命名为国家级双拥模范县。精心组织开展严打整治斗争，不断完善社会治安防控体系，人民群众安全感明显增强。扶沟县被评为全省平安建设先进县。建立健全应急机制，深入排查化解矛盾纠纷，在全国“两会”和奥运会等重要活动期间，没有发生赴京、赴省非正常上访和大规模集体上访事件。商水县、太康县被评为全省大接访工作先进县。深入开展民族团结进步教育活动，民族宗教工作得到进一步加强。

　　各位代表，回顾一年来在国内外环境复杂多变、约束因素不断增加、区域竞争日益加剧情况下的工作实践，我们深深体会到，要实现周口追赶型跨越式发展，必须把思想统一到市委的重大决策上来，坚持市委确定的经济发展方略和产业发展方向不动摇，始终按照市委的统一部署，一以贯之地推进政府各项工作；必须把精力集中到项目建设上来，坚持招商引资不动摇，始终以项目建设为核心，实施项目带动战略，不断推进园区建设，做大支柱产业，为长远发展打基础、增后劲；必须把智慧凝聚到改革创新上来，坚持以经济建设为中心不动摇，始终围绕科学发展这个主题，不断突破各种思想障碍，创新体制机制，切实增强经济社会发展的动力和活力；必须把成果惠及到广大人民群众利益上来，坚持紧贴民心不动摇，始终从人民群众最关心、最直接、最现实的利益问题入手，尽心竭力为群众办实事、做好事、解难事，让人民群众充分享受改革发展成果；必须把行动落实到真抓实干上来，坚持求真务实工作作风不动摇，始终按照“两转两提”、“提速增效”要求，在工作中切实转变职能、提高效率，明确目标、强化责任，确保制定的各项目标任务、部署的每项工作都能落到实处、取得实效。

　　各位代表，一年来取得的成绩来之不易。这是全市上下和衷共济、共渡时艰的结果，凝聚着全市人民的心血和智慧，得益于社会各界的大力支持和帮助。在此，我代表市人民政府，向工作在全市各条战线上的工人、农民、知识分子、各级干部、离退休老同志、人民解放军指战员、武警消防官兵、公安司法干警，致以崇高的敬意！向给予政府工作大力支持的人大代表和政协委员，向各民主党派、工商联、各人民团体和各族、各界人士，向驻周单位及所有关心、支持我市现代化建设的同志们、朋友们，表示衷心的感谢！

　　在肯定成绩的同时，我们也清醒地看到，我市经济社会发展还存在一些困难和问题。主要是：受国际金融危机冲击和国内经济增长减速影响，经济增长速度放缓，发展压力增大，有些重点项目进展不理想，“双十五工程”中个别项目没有完成年度任务；农业基础设施仍然薄弱，劳务输出和农民工就业形势严峻，保持农民增收难度加大；产业结构调整和发展方式转变任务艰巨，资金、人才、技术等要素瓶颈制约更加突出；城市建设和管理与市民的期望还存在一定的差距；环境保护、节能降耗和污染减排任务较重；安全生产形势仍较严峻；有些职能部门作风不实，官僚主义、形式主义还不同程度地存在，发展环境有待进一步优化。对此，我们必须高度重视，采取有效措施，认真加以解决。

　　二、２００９年政府工作的总体要求和奋斗目标

　　各位代表，２００９年，我们面临的是一个希望与困难同在、机遇与挑战并存、机遇大于挑战、总体上有利于加快发展的环境。一方面，国际金融危机尚未见底，金融危机的影响正逐步从金融领域向实体经济、从沿海向内地、从中小企业向大企业、从出口行业向其他行业扩散，经济增长明显减速，宏观经济形势对我市发展的影响不断加重。另一方面，国家采取扩大内需、促进增长的十项政策措施，４个重点投资领域两年投资规模达４万亿元，省里实施“８５１１”投资促进计划，国际国内区域合作、产业调整和转移步伐加快，给我市带来奋力追赶的难得机遇。我们一定要全面辩证地看待面临的形势，既要未雨绸缪、及早谋划，把困难估计得更充分一些，把应对措施考虑得更周密一些，更要坚定信心、“危”中求“机”，主动从变化的形势中捕捉和把握难得的发展机遇，在逆境中发现和培育有利因素，保持经济社会持续平稳较快发展的良好态势。

　　今年政府工作的总体要求是：以党的十七大和十七届三中全会精神为指导，深入学习实践科学发展观，认真贯彻中央经济工作会议、省委八届九次全会和市委二届七次全会精神，准确把握国家宏观调控政策，以保持经济平稳较快增长为首要任务，加快改革创新和转变发展方式，突出项目建设和产业培育，全力推进“双十五工程”，加强资源节约和环境保护，协调发展各项社会事业，切实抓好“十件实事”，积极改善和保障民生，促进经济社会又好又快发展，奋力开创追赶跨越新局面。

　　今年经济社会发展的主要预期目标是：生产总值增长１１％；地方财政一般预算收入增长１２％；全社会固定资产投资增长２０％；社会消费品零售总额增长１４％；出口总额增长１０％，实际利用外资增长１３％；居民消费价格总水平涨幅控制在４％左右；城镇居民人均可支配收入增长７％，农民人均纯收入增长６％；新增城镇就业人数６万人，城镇登记失业率控制在４．６％以内；人口自然增长率控制在６．５‰。以下；万元ＧＤＰ能耗降低４．４％；化学需氧量、二氧化硫排放量分别下降３．３％和０．７％。

　　各位代表，按照上述总体要求和预期目标，做好今年政府工作，必须深入贯彻落实科学发展观，坚持开放先导、创新驱动，大抓项目、抓大项目，努力在招商引资和项目建设上有新作为；坚持抢抓机遇、加快发展，进一步强化工业主导地位，努力在促进工业经济平稳较快增长上有新作为；坚持统筹城乡、协调发展，围绕稳粮、增收、强基础，努力在促进农业稳定发展、农民持续增收上有新作为；坚持节约资源、保护环境，推进节能减排，努力在生态文明建设上有新作为；坚持以人为本、促进和谐，认真解决关系人民群众切身利益的突出问题，努力在改善民生上有新作为；坚持求真务实、真抓实干，切实提高推动科学发展的能力和水平，努力在政府自身建设上有新作为，不断开创周口各项工作的新局面。

　　三、千方百计保持经济平稳较快发展

　　国际金融危机影响日趋严重，周口经济发展面临诸多不确定因素。我们必须完善工作思路，创新工作机制，总揽全局抓大事，突出重点求突破，积极主动地打好应对危机与挑战这场硬仗，保持周口经济平稳较快发展。

　　（一）突出重大项目建设，保持有效投资增长

　　实现周口追赶型跨越式发展，项目是关键，投资是保证。今年是项目推进年，我市围绕国家和省投资重点，筛选建设项目１００个，总投资２２０亿元。在整体推进的同时，必须突出抓好事关经济发展、具有重大支撑和带动作用的重点项目，全力推进“双十五工程”。

　　——全力推进十五项重点工业项目。十五项重点工业项目，总投资６５．３亿元，今年投资２８．４３亿元。

　　１．总投资８亿元，今年投资５亿元，开工建设河南大用集团西华分公司肉鸡产业化项目；

　　２．总投资５．８亿元，今年投资２．５亿元，开工建设鑫达纺织印染有限公司扩建项目；

　　３．总投资４亿元，今年投资１亿元，开工建设雨润食品加工有限公司肉制品加工项目；

　　４．总投资１．６亿元，今年投资５０００万元，开工建设扶沟惠丰鞋业有限公司制鞋项目；

　　５．总投资１．３亿元，今年投资８８００万元，开工建设亚华安全玻璃有限公司汽车安全玻璃项目；

　　６．总投资２６．３亿元，今年投资５亿元，继续建设周口隆达热电厂２×３０万千瓦机组项目；

　　７．总投资５．７亿元，今年投资３．９亿元，继续建设宏亿木业有限公司中高密度纤维板项目；

　　８．总投资２．２亿元，今年完成全部投资，建成天瑞集团周口水泥有限公司二期生产项目；

　　９．总投资２．１亿元，今年完成全部投资，建成财鑫集团年产３万吨生物低碳多元醇项目；

　　１０．总投资２亿元，今年投资９０００万元，建成裕达食品股份有限公司豆制品加工项目；

　　１１．总投资１．８亿元，今年投资４５００万元，建成五得利集团周口面粉有限公司日加工小麦２４００吨项目；

　　１２．总投资１．２亿元，今年完成全部投资，建成银丰塑料有限公司年产３万吨农用塑料棚膜项目；

　　１３．总投资１．２亿元，今年投资７０００万元，建成河南阳光电缆有限公司年产１００纤芯万公里光缆项目；

　　１４．总投资１．１亿元，今年完成全部投资，建成益海（周口）粮油工业有限公司日产１０００吨榨油加工项目；

　　１５．总投资１亿元，今年完成全部投资，建成河南莲花味精股份有限公司小麦淀粉制糖生产项目。

　　——全力推进十五项重点基础设施项目。十五项重点基础设施项目，总投资７９．７６亿元，今年投资２８．３６亿元。

　　１．总投资６０亿元，今年投资２０亿元，开工建设周口漯阜铁路电气化改造（周口段）项目；

　　２．总投资４．７７亿元，今年投资１亿元，开工建设涡河航运开发建设一期(周口段)项目；

　　３．总投资３．３亿元，今年投资７０００万元，加快市沙南污水处理二期工程项目建设；

　　４．总投资３．２亿元，今年投资１．２亿元，加快市职业教育示范基地项目建设；

　　５．总投资１．０２亿元，今年投资７０００万元，加快沙颍河大庆路桥项目建设；

　　６．总投资８９００万元，今年投资５９００万元，加快市体育馆项目建设；

　　７．总投资３４００万元，今年投资２０００万元，加快市科技馆项目建设；

　　８．总投资１．９８亿元，今年投资９０００万元，建成市焚烧式垃圾处理项目；

　　９．总投资１．６亿元，今年投资７０００万元，建成文昌大道西段改造项目；

　　１０．总投资１．１９亿元，今年投资１亿元，建成市文化艺术中心项目；

　　１１．总投资８３６１万元，今年完成全部投资，建成周口大道拓宽改造项目；

　　１２．总投资６３６３万元，今年投资５３６３万元，建成贾鲁河建设路桥项目；

　　１３．加快周口火车站搬迁项目初步设计、征地拆迁等前期工作，争取年内开工建设；

　　１４．加快周口客运总站建设项目规划选址、征地拆迁、项目审批等前期工作，争取尽早开工；

　　１５．加快周口港口物流园区建设项目规划、环境和洪水评价、可研报告、初步设计等前期工作，争取尽早开工。

　　对这些关系全局的重点项目，在配置土地资源、保证环境准入、办理各项手续等方面给予政策优惠。严格实行重点项目责任制，重点项目所在县市区和市直有关单位，切实加强项目管理，搞好项目服务，做到一个项目、一套班子、一抓到底。下大力气抓好郸（城）淮（阳）一级公路、商水瑞肯国际有限公司鞋业生产加工、周口５００千伏输变电项目等一批未列入“双十五工程”的建设项目，及时解决项目推进中遇到的困难和问题，使我市的项目建设取得显著成效。

　　（二）大力发展现代农业，开创“三农”工作新局面

　　做好今年农业农村工作，对于应对危机、保持经济平稳较快发展具有特殊重要意义。必须高度重视“三农”工作，加快发展现代农业，扎实推进新农村建设。

　　进一步稳定和提高粮食综合生产能力。认真落实《国家粮食战略工程河南核心区建设规划》，积极组织实施优质粮食产业工程、科技支撑体系建设、粮食科技研究与应用工程等农业项目，加快高产优质农作物新品种的推广应用步伐，确保粮食作物的优良品种覆盖率达到１００％。开展全面高产攻关，努力提高秋粮单产水平。加快推进农业机械化，搞好农田水利基本建设，开工建设世行贷款平原洼地治理项目，完成农业综合开发土地治理３０万亩，加大中小河流治理和病险涵闸除险加固力度，增加水利建设投入，不断改善农业生产条件。加强人工影响天气管理，增强气象防灾减灾能力。强化耕地保护，粮食播种面积稳定在１６００万亩左右，粮食总产稳定在１４０亿斤左右。

　　加快推进现代农业示范区建设。立足农业大市实际，突出地方特色，用工业理念发展现代农业，从土地流转、农业新技术推广、社会化服务等环节入手，建设现代农业示范区，力争通过几年的努力，每个县市区都建成１个集科技先进、特色明显、产业高效、绿色生态于一体的现代农业示范区。

　　大力发展现代畜牧业。把建设和发展标准化规模饲养场（小区）作为转变畜牧业生产方式的重点，新建标准化养殖场（小区）１８０个以上，改造和规范标准化养殖场（小区）１６０个。组建年出栏２０万头以上的生猪养殖集团２个，年出栏２０００万只以上的养禽集团２个。积极组织开展无公害畜产品产地认定和产品认证工作，今年全市符合条件的规模养殖企业全部通过无公害畜产品产地认定。进一步完善基层动物疫病防控体系，在全市建立２６０个市级疫情监测点，市动物疫病监测检验中心通过认证并投入使用。加强畜产品质量安全监管，提高质量安全水平。

　　切实抓好农业产业化经营。加大种植业结构调整力度，积极发展专用粮食、食用菌等优质、高产、高效农业，大力发展名、特、优、新农产品。坚定不移地实行龙头带动战略，培育年销售收入超亿元畜产品加工企业５家，其中１０亿元以上企业３家；重点实施“５０家龙头企业培育工程”、“５０个标准化生产基地建设工程”和“５０家示范专业合作社扶持工程”，培育农业产业化龙头企业集群。落实各项优惠政策，加大扶持力度，支持龙头企业做大做强。引导和支持龙头企业采取订单农业、入股分红、利润返还等方式，密切并规范同农民、农业生产的联系，增加农民收入。积极发展农民专业合作经济组织，年内力争达到１０００家。

　　改善农民生产生活条件。抓住国家新一轮鼓励和促进农村经济发展机遇，搞好新农村建设规划，积极争取项目和资金，加强农业和农村基础设施建设，有效改善环境卫生状况和村容村貌。改造县乡村道路１０００公里，开工建设县乡公路大中危桥９２座，不断完善农村公路网络。争取建成投运扶沟、太康、商水等７个县级客运站。完成１５０个贫困村扶贫开发整村推进任务。解决４０万人饮水安全问题。新增沼气用户８万户。创建１５个省级生态文明村。搞好“万村千乡市场工程”。完成２００个行政村的农民体育健身工程。改造扩建３３个乡镇文化站，建设２６０个农村书屋。全年送戏下乡不少于２０００场，为所有行政村每月公益性放映电影１场。

　　（三）强化工业主导地位，促进工业经济平稳较快增长

　　保增长首先必须保工业。坚持一手抓新兴产业培育，一手抓传统产业改造，加大资金支持和政策激励，大力推动结构调整和产业升级，努力实现工业经济的新跨越。

　　精心培育大型骨干和优势中小企业。紧紧抓住国家扩大投资、增值税转型、国际原材料和能源价格明显下降的有利条件，落实和完善分类指导措施，通过企业战略重组，加快产业结构调整，发展上下游关联企业，培育在省内外有影响、具有辐射带动作用的龙头企业，力争全年规模以上工业企业达到９００家；抓好现有３０户重点企业和１０户高成长性企业，强化梯队建设，实行动态管理，加大扶持力度，促其做大做强。全力支持金丹乳酸、金丝猴集团、四方药业等企业上市直接融资。用好中小企业发展专项资金，对发展潜力大的中小企业给予支持。３月底以前，各县市区都要建立中小企业贷款担保机构，足额安排配套资金。在现有中小企业创业基地的基础上，争取建成国家级中小企业创业基地１～２个、省级中小企业创业基地３～４个。建立健全技术支持服务体系，争取国家级中小企业公共服务平台扶持项目１个。

　　积极发展壮大支柱产业。编制食品、纺织服装和医药化工产业发展规划，巩固发展电力、机械、化工等基础产业和皮革皮毛等特色产业。食品加工业主要是拉长产业链条，发展终端产品。重点抓好莲花味精、益海（周口）粮油、大用邦杰等龙头企业和优势产品，提升粮油加工和肉食产品加工两大优势产业，做强做精食用油、肉制品和面粉加工产品链，培育壮大乳制品、果蔬、休闲食品等高成长性产业，推动食品工业由群体优势向“品牌＋规模”优势转化。纺织服装业主要是完善产业链条，淘汰落后产能，促进产业升级。继续支持鑫达纺织、中方纺织、昌茂棉纺、项城棉纺等纺织企业扩大产能和提升品质，加快香港帝域集团服装加工基地、广州森仕时装（周口）有限公司服装加工等项目建设进度，大力引进开发符合环保要求、有一定规模的印染、织布企业和知名服装品牌生产，提升产品附加值。医药化工业主要是高新技术的引进和使用，提高市场竞争力。以辅仁药业、财鑫集团、四方药业、龙都药业等企业和优势产品为龙头，重点发展生物医药、现代中药、新型化学合成剂等产品，增强自主研发能力，打造有市场影响力的中成药品牌，实现产业和市场的良性互动。

　　加快工业园区建设。做好工业园区产业规划，明确列入省总体规划的１２个工业园区的功能定位，优化园区布局，防止无序竞争，着力解决园区同质化发展问题。创新园区运作机制，优化服务流程。加大土地储备力度，完善园区基础设施配套，增强园区承载功能。抓住省政府扶持产业集聚区发展的政策机遇，加快鹿邑尾毛、太康锅炉、淮阳塑编、沈丘造纸网业、项城秣陵制鞋等特色产业集聚区的发展，引导同类产业项目集中摆放，相关产业集中发展，资源科学利用，促进园区成为资源节约型、环境友好型产业的集聚区、科技创新的先导区。

　　增强自主创新能力。加强与郑州大学、河南工业大学的科技合作，鼓励企业与高校院所合作建立独立建制的研究所，增加技改投入，培育一批拥有自主知识产权、具有核心竞争力的高新技术企业，争取今年有２～３家企业被评定为省高新技术企业。抓好企业工程中心、重点实验室等研发机构建设，争取今年再建工程技术中心１０家。引导企业树立强烈的创新意识、品牌意识，使更多的产品跨入全省、全国的名牌序列。强化企业管理，引进先进的管理和工艺流程，提高企业整体素质和市场竞争能力。

　　扎实开展“企业服务年”活动。在当前困难形势下，企业最需要政府雪中送炭、添薪助暖，各级政府必须围绕企业需求抓好经济调节，建立完善经济运行组织协调机制和服务企业发展的工作体系。采取建立政府班子联系企业制度、抓重点企业带一般企业、召开协调会和办公会、帮助企业谋划项目、支持企业争取上级资金、减轻企业负担等方式，最大限度减轻外部环境变化对企业生产经营造成的冲击，努力保持工业经济平稳较快增长。

　　（四）加快城镇化进程，统筹城乡协调发展

　　积极推进中心城区与县域城镇、居民点建设，加快周商、周淮一体化进程，促进城市空间布局优化、承载能力提升、服务功能完善、特色魅力彰显。大力发展县域经济，实现城乡统筹发展。

　　增强中心城市承载力和吸纳力。抓住漯阜铁路电气化复线改造机遇，调整完善中心城区规划，编制旧城区改造规划，做好开发区规划调整和部分总规修编，加快周口港口物流园区建设规划，抓好中心城区的地下管网普查工作，用规划引领城市建设。加快五一路南段、工农路南段、南环路东段、东三路、东四路等道路建设，争取开工建设沙北客运站，启动部分棚户区和“城中村”改造，建设东新区公厕、垃圾中转站等公共设施，积极筹建东新区水厂，进一步完善基础设施。大力发展以服务业为主的城市特色产业，巩固提升商贸流通、交通运输、餐饮住宿等传统服务产业，加快发展中介服务、社区服务、金融保险等新兴服务业，着力发展建筑业和房地产业，加快房地产项目开发建设。以创建省级卫生城为目标，不断创新城管理念，推行城市网格化管理和精细化管理，将城市管理覆盖、延伸到各个社区和街道。充分发挥川汇区、开发区、东新区的作用，切实做到统一领导、属地管理、部门协调、权责分明。着力解决城市违章搭建、占道经营、交通秩序混乱、乱倒垃圾等难点问题，整治市容市貌，搞好美化亮化，提升城市品位。

　　办好中心城区１０件实事：１．硬化建成区（不含改造区）全部小街小巷；２．实现城市低保户价格补贴全覆盖；３．改造西大街低洼区排水系统；４．改造供水低压区９个；５．新增天然气用户１万户；６．亮化次干道５条；７．建设东新区幼儿园１所；８．建设社区卫生服务中心（站）３个；９．新建或改造公厕１０个；１０．新建或改造垃圾中转站５座。

　　加快县城和小城镇建设。做好《城乡规划法》的宣传落实工作，加强村镇的规划、建设和管理。鼓励基础条件好、产业支撑强、辐射半径大的县城，发展成为区域中心县城。大力发展专业镇和沿线、沿边城镇带，突出抓好国家、省、市确定的３９个试点镇、重点镇的扩容提质，创建２个省级环境优美小城镇。坚持把县城、小城镇、居民点建设与市场建设、产业集聚发展结合起来，增强城镇综合经济实力和辐射力，引导人口向城镇发展、产业向园区集中，以人口和产业集聚带动县城规模扩大和中心城镇发展，形成优势互补、各具特色的中小城市格局。力争城镇化率达到２９％。

　　发展壮大县域经济。紧紧围绕“农业增收、工业增效、财政增长、后劲增强”这个目标，用好国家进一步加大“三农”投入和省支持黄淮四市发展政策，采取加快工业发展、扩大招商引资、推进项目建设、壮大民营经济、创新体制机制、优化发展环境六项举措，因地制宜，发挥优势，形成特色鲜明的县域经济发展格局，不断提升各县市在全省整体排名中的位次。把县域经济发展的主要指标列为考核考评领导干部政绩的重要内容，建立健全考核考评办法和奖惩机制，并将县域经济发展情况按年度定期向社会公布。

　　（五）围绕建设文化强市，实现文化旅游产业发展新突破

　　发挥周口文化底蕴丰厚、生态良好的优势，坚持文化旅游两篇文章一起做，努力把文化旅游资源优势转化为经济发展优势。

　　发展文化事业。鼓励支持文艺创作，着力打造一批具有周口特色的文艺精品；举办周口市第三届小戏小品大赛。发挥１５个数字电影广场的功能，实现公益性电影放映常态化。加强文物资源和非物质文化遗产保护、开发及利用，启动周口关帝庙三期修缮工程，抓好平粮台古城遗址保护与开发，完成我市第三次全国文物普查田野调查工作。重视周口越调、太康道情、太昊伏羲祭典和老子生日祭典的保护和发展。挖掘整理文化资源，申报国家级不同类型的“民间艺术之乡”。大力开展图书、音像、网吧等行业专项整治，净化文化市场。

　　壮大文化产业。全面落实文化产业扶持政策，大力发展网络服务、广告会展、文化创意等新兴文化产业，支持文化娱乐、休闲健身和艺术培训等社会文化产业发展。整合书画经营、杂技培训与演出等文化资源，进一步打造公益性文化品牌“周末一元剧场”。大力发展特色文化产业集群，建设汝阳刘笔业文化产品生产和周口关帝庙文化产品经营２个文化产业示范园区，加快淮阳文化改革发展试验区建设。利用周口日报社、周口电台电视台等现有资源，培育一批市场占有率高、品牌响、扩张能力强的文化产业集团，切实增强文化产业的竞争力。

　　做强旅游产业。大力发展淮阳农家乐旅游，做好龙湖度假休闲游，鹿邑李姓、淮阳陈姓姓氏寻根游。举办周口人游周口系列活动，拓展本地游客源市场。加快太昊陵、老子故里、关帝庙、女娲城、袁世凯故居、叶氏庄园、中原民俗园、吉鸿昌纪念馆等景区的配套建设与管理，不断提高景区内涵和品位。继续以对接开封、对接曲阜为主要促销手段，加大宣传推介力度，加强区域合作，形成“周—淮—鹿、周—项—淮、周—西—扶”三条旅游精品线路。积极争取上级旅游部门支持，加强与国内外旅行社的联系与合作，使周口文化旅游融入全省、全国旅游大格局中，吸引更多国内外游客来周口观光旅游。

　　（六）坚持深化改革，全面提升对内对外开放水平

　　深化改革、扩大开放是扩内需、保增长、调结构、促转型的动力，必须稳步务实地推进重点领域和关键环节的改革，全面扩大对外开放和区域合作，增强经济社会发展的动力和活力。

　　全面推进改革创新。稳定和完善农村基本经营制度，保持农村现有土地承包关系稳定并长久不变；鼓励农民按照依法、自愿、有偿原则，以转包、出租、互换、转让、股份合作等多种形式流转土地承包经营权，推进土地适度规模经营，所有县和部分乡建立土地流转管理和服务机构；进行城乡建设用地存量挖潜改造，实行最严格的土地管理制度和耕地保护制度；大力推进集体林权改革；建立运转灵活、服务高效的新型农村体制机制，解放和发展农村生产力。深化国企改革，巩固国企改革成果；加快宋河酒厂改制进程；推进商贸、供销、物资等流通企业改革，不断增强发展活力。进一步强化财税职能，完善事权与财权相匹配的财税体制。深化国库集中收付、政府采购和投资评审管理制度改革，加强财政资金筹集、分配、管理的全过程监督，严格控制一般性支出，公务经费支出实行零增长，提高财政运行质量和资金使用效益。改善和创新农村金融服务，充分发挥涉农金融机构的作用，完善支持农民小额贷款办法；积极推进农信社改革成为农村商业银行，城市信用社年内成功组建为城市商业银行。加快中小学人事制度改革步伐，完成５４所学校的试点改革任务。积极稳妥推进政府机构改革，上半年完成市级政府机构改革，年底前完成县级政府机构改革。同时，加快事业单位分类改革。

　　不断提高开放型经济水平。坚持专业化、市场化、园区化、产业化招商引资的路子，突出引进战略投资者和大项目。优化招商引资项目库，增强项目投资吸引力。通过参加省政府组织的各类经贸合作洽谈会等重大经贸活动，集中发布项目，强势推介周口。加大工业、基础设施和服务业项目的招商力度，不断拓宽引资领域。密切同长三角、珠三角、环渤海、港澳台等重点地区的联系，充分发挥人文、资源、区位等优势，加快承接国际及沿海发达地区产业转移。强化软环境建设，努力营造“亲商、富商、安商”的发展氛围，确保完成实际利用省外资金１３８亿元和外商直接投资 １．２亿美元的目标任务。推动莲花味精、金丹乳酸等２０家重点出口企业创建出口品牌，不断提高企业的出口竞争力和出口效益，努力完成４．５亿美元的外贸进出口任务。

　　大力推进全民创业。认真贯彻落实全市全民创业暨农民工工作会议精神，充分利用现有存量用地、闲置厂房、专业化市场等适合创业的场所，因地制宜建设具有滚动孵化功能的创业孵化园区。鼓励返乡农民工、大中专毕业生和其他新增就业群体等各类人员自主创业和从事个体经营，积极引进市外投资主体来周创业，发挥大中型企业在创业中的引领带动作用，支持其不断开拓新的创业领域。加大银行信贷和财政支持创业力度，小额担保贷款额度可达５万元，新招用登记失业人员达到一定比例企业，贷款额度可达３００万元；市财政每年在预算内安排创业引导资金５００万元，县市区财政安排２００万元。

建立全民创业指导服务组织，鼓励社会各类服务机构开展多种形式的创业服务活动。从今年起，组织实施“万人创业计划”，每年组织１万人开展创业活动，力争５年内实现全市私营企业由０．８７万户增加到１．９万户，个体工商户由１０．８万户增加到１４．６万户，并由此带动近３０万人就业。

　　（七）加强节能减排和生态建设，转变经济发展方式

　　加快形成节约能源资源、保护生态环境的经济发展方式，推进资源节约型、环境友好型社会建设。

　　强力推进节能减排。认真落实国家和省节能减排政策，严格实行节能减排问责制和“一票否决”制，打好节能减排攻坚战。严格执行能耗准入标准，加快节能减排技术进步。加强建筑节能，大力发展新型墙材生产企业，新建公共建筑和住宅分别达到节能５０％和６５％的要求。大力推进公共机构节能，积极发展太阳能等清洁再生能源。推广农业节水技术，改造城市供水管网，节约生产生活用水。加大城市污水管网配套建设力度，提高污水处理厂运营水平。坚持不懈地抓好贾鲁河、沙颍河等重点流域和８家重点企业的环境综合整治，确保河流断面水质持续改善。加大对食品、医药、化工等氨氮排放量较大行业的治理力度，在化工、电力、酿造等行业开展大气污染防治工作。继续推进农村环境保护工作，完成２家规模化畜禽养殖场污染治理示范工程。

　　加强林业生态建设。认真组织实施《周口市林业生态建设规划》和《周口国家现代林业示范市建设规划》，将太康、淮阳和郸城建成省级林业生态县。以平原林业生态区、城市、村镇和生态廊道网络为重点，抓好造林绿化，植树４６万亩，完成１２４个乡镇１３２８个村７．９万亩的绿化工程、１１．６万亩的农田防护林体系建设、１．１万亩防沙治沙工程、５．６万亩的生态廊道网络建设。搞好中心城市美化绿化，进一步提高全市绿化整体水平。

　　大力发展循环经济。用好国家和省扶持循环经济发展政策，突出抓好沈丘县付井镇国家级循环经济试点单位建设，全面推进电力、食品、化工等行业及农业、林业、畜牧业等领域的循环经济发展。鼓励企业加大环保投入力度，完善环保设施。加快推进莲花、财鑫、天瑞、国能、大华等循环型企业发展，着力培育一批再生资源循环利用企业，大力推进农作物秸秆和工业废渣、废水、废气等废旧资源再生利用。

　　四、积极促进社会和谐

　　越是经济困难时期，越要切实关心百姓的衣食住行、安危冷暖，注重认真解决涉及群众切身利益的问题，高度重视维护社会和谐稳定。

　　（一）千方百计扩大就业。实施更加积极的就业政策，发挥政府投资和重大项目带动就业的作用，发展劳动密集型产业、服务业和中小企业，千方百计增加就业岗位。继续延长“４０５０”人员享受公益性岗位就业的各项补贴政策，动态消除“零就业家庭”。支持困难企业采取在岗培训、轮班工作、协商减薪等办法，尽量不裁员或少裁员。增加就业再就业小额贷款，建立动态管理、动态援助机制，加强对城镇特困家庭成员、连续失业１年以上人员、残疾人员等困难群体的就业援助。实现下岗失业人员再就业３万人。鼓励大中专毕业生转变择业观念，到基层、中小企业和非公有制企业就业，做好复转军人就业安置工作。实施“阳光工程”培训２．５万人、扶贫培训“雨露计划”２万人和农村劳动力技能培训计划，新增１０万农村富余劳动力转移就业。

　　（二）继续完善社会保障体系。进一步扩大养老保险覆盖范围，确保养老金按时足额发放。完善失业保险制度，确保失业保险金按时足额支付。认真解决进城务工人员和失地农民的社会保障等问题。将在校大学生全部纳入城镇居民医保范围，进一步提高城镇居民医保和新农合的参保率。及时为依法关闭破产企业退休人员办理医保接续。扩大工伤保险覆盖面，力争参保人数达到１６万人。完善城乡社会救助体系和优抚保障制度，积极发展社会福利和慈善事业，开展多种形式的捐助和帮扶活动。巩固农村敬老院建设成果，提高五保集中供养率，将年供养标准由１４００元提高到１６００元。加大保障性住房投资建设力度，开工建设经济适用房１２万平方米、廉租房３．５万平方米，为城市低收入家庭提供１７００套经济适用房和７００套廉租住房。

　　（三）积极发展各项社会事业。继续解决中心城区和县城大班额问题。将农村中小学义务教育学校生均公用经费标准分别由３７５元和２３０元提高到５００元和３００元，继续对农村义务教育阶段学生全部免除学杂费、免费提供教科书，补助农村寄宿制学校家庭经济困难学生生活费。继续免除城市义务教育阶段学生学杂费。完成２６所农村初中校舍改造工程和１８．１万平方米的农村中小学校舍维修改造工程。大力推进职业教育攻坚计划，完成沈丘县职教中心综合实训楼工程和特殊学校建设工程。抓住河南创立国家职业教育改革实验区的机遇，筛选争取一批职教项目。推进周口幼师、周口卫校、周口科技职业学院入驻东新区职教示范基地工作，开展争创省级职业教育强县活动。加强教师队伍建设，启动新一轮中小学教师培训计划。大力实施普通高中质量提升工程，力争全市高考再上新台阶。加强各级疾病预防控制机构实验室装备建设，完善疫情监测网络。健全全市突发公共卫生事件应急体系。加强城市社区卫生服务体系建设，为城市居民提供基本医疗服务。建设１５００个标准化村卫生室，为乡镇卫生院配备基本医疗设备，完成３９个薄弱乡镇卫生院改造，新建扩建４３个乡镇计生服务站。创建“白内障无障碍市”，免费实施１万例贫困白内障患者复明手术。进一步抓好艾滋病救治救助工作，全面落实结核病“三免费”政策。扩大免疫规划，在使用５种国家免疫规划疫苗预防７种传染病的基础上，扩展到使用９种疫苗预防１２种传染病。严格计划生育管理，稳定低生育水平。综合治理出生人口性别比偏高问题。完善计划生育利益导向机制，农村部分计划生育家庭奖励扶助标准由每人每年６００元提高到８４０元。积极应对人口老龄化。广泛开展全民健身活动，促进群众体育和竞技体育共同发展。

　　（四）进一步做好安全生产和食品药品安全工作。全面落实安全生产责任制，强化行政问责和责任追究。深入开展安全生产隐患排查治理工作，着力抓好交通运输、烟花爆竹、危险化学品、建筑工程等重点行业和重点企业的安全治理，抓好商场、学校、娱乐场所等公共聚集地的消防工作，坚决遏制重特大安全事故发生。建立健全农产品质量安全检验监测体系，建设市级食品药品检验所和９个县级农产品质量安全检验检测站；努力创建食品安全优秀城市，完成１至２个县（市）省级食品安全示范县创建任务，培育一批示范乡镇和示范企业。以整顿规范小麦粉、食用油、奶制品、配制酒为重点，全面排查安全隐患，强化环节监管和企业责任，坚决杜绝不合格产品进入市场。全市城市、农村食品市场抽验合格率比上年分别提高２至３个百分点。巩固完善农村药品供应网和监督网建设，鼓励药品连锁和集中配送向农村发展。力争药品抽验合格率达到９８％以上。

　　（五）切实加强精神文明建设。深入开展学习实践科学发展观活动，加强社会主义核心价值体系建设，大力弘扬抗震救灾精神，在全社会树立知荣辱、讲正气、树新风、促和谐的新风尚。深化爱国主义、集体主义、社会主义教育，加强社会公德、职业道德、家庭美德和个人品德教育，广泛开展群众性精神文明创建活动，积极倡导科学、健康、文明的生活方式。加强未成年人思想道德建设和大学生思想政治教育工作。深入开展创建文明城市工作，广泛开展文明行业、文明单位、文明社区和文明村户创建活动。进一步组织好文化、科技、法律、卫生“四下乡”和科技、文体、法律、卫生“四进社区”活动，提高全民科学文化素质。

　　（六）全力维护社会和谐稳定。在完善领导干部大接访的基础上，进一步搞好信访工作，健全矛盾纠纷排查化解机制，妥善处理群众反映强烈的问题，坚决纠正损害群众利益的行为。加强应急体系建设，提高应对各类突发公共事件的能力。继续推进“五五”普法工作。加强法律援助，依法保障公民特别是妇女、未成年人和残疾人的合法权益。深入开展“平安周口”建设工作，加强社会治安综合治理，依法防范和打击各类违法犯罪活动，增强人民群众安全感。发挥工会、共青团、妇联等人民团体的桥梁纽带作用，积极参与社会管理和公共服务。加强社区建设，完善村民自治。加强国防教育，增强全民国防意识。支持驻周部队和民兵预备役建设，继续做好人民防空和“双拥”工作，鼓励县市努力争创省级“双拥”模范城（县），进一步密切军政军民关系。广泛开展民族团结进步活动，巩固和发展平等团结、互助和谐的社会主义民族关系。全面贯彻党的宗教工作基本方针，发挥宗教界人士和信教群众在促进经济社会发展中的积极作用。加强统计工作，抓好第二次经济普查工作。努力做好审计、物价、气象、地震、档案、外事、侨务、史志等工作。继续抓好对口支援江油市大堰乡、西屏乡灾后重建工作，按期完成援建任务。

　　五、努力建设人民满意政府

　　２００９年是政府管理创新年。面对异常严峻的经济形势、繁重的工作任务和前所未有的挑战，各级政府要进一步解放思想，围绕“两转两提”、“提速增效”，按照科学化、规范化的要求，完善规则，建章立制，使政府工作规范有序运行；通过改革创新、作风转变，努力提高行政能力和服务水平，不断开创政府工作新局面。

　　（一）转变政府职能，提高服务水平。按照“精简、统一、效能”的原则与“决策、执行、监督”相协调的要求，加快推进政府职能转变，不断规范政府职能定位。发挥好经济调节、市场监管的职能作用，积极应对国际金融危机的影响；更加注重履行社会管理和公共服务职能，扩大政府公共服务的覆盖范围，提高公共服务的供给能力和质量，着力为市场主体创造良好的发展环境，确保服务到位。加快推进政企分开、政事分开、政资分开、政府与市场中介组织分开，支持社会中介组织、行业协会在社会管理和公共服务中发挥更大的作用。进一步加强行政服务中心建设，切实增强部门服务意识，转变思维方式，强化优质服务，尽心竭力为群众办好事、解难事，把爱民之情、为民之心、惜民之意体现在政策执行上，落实在解决具体困难上，践行在为民办实事上。

　　（二）坚持依法行政，规范政府行为。认真贯彻落实《国务院关于加强市县依法行政的决定》，大力增强和提高市县行政机关工作人员依法行政的意识和能力，严格实行行政执法责任制和执法过错追究制，坚决制止“不作为”和“乱作为”等行为。积极推进科学决策和民主决策，完善听证制度和重大决策前的专家论证、群众评议制度，优化政府决策规则和程序，使决策更加符合客观实际，更加符合群众的愿望和利益。大力实施政务公开，拓宽新闻舆论监督、社会公众监督等渠道，构建权力阳光运行机制，扩大人民群众的知情权、参与权、监督权，使政府工作更加透明。加强民主政治建设，认真贯彻落实市委的各项决策，自觉接受市人大的法律监督、工作监督和政协的民主监督，虚心听取批评、意见和建议，充分发挥各民主党派、工商联、无党派人士的参政议政作用，积极支持工会、共青团、妇联等群众团体开展工作。

　　（三）强化效能建设，狠抓工作落实。大兴真抓实干、雷厉风行的工作作风，进一步提高政策执行力和工作落实力。对重大决策和部署、重大投资和项目，建立健全抓落实的领导机制和工作机制，加强行政效能监察，确保各项工作取得实实在在的成效。继续广泛开展民主评议市直单位及其重点科室（二级机构）活动，严肃查处涉企“四乱”行为，做到政令畅通、令行禁止，进一步优化经济发展环境。切实改变工作作风，深入实际、深入基层、深入群众，开展调查研究，及时发现问题和解决问题，坚决防止形式主义、官僚主义，坚决摆脱文山会海。市政府带头精减会议、文件，严格控制各类检查、评比，精简、务实地举办节庆活动。各级政府部门特别是领导干部要腾出更多的精力谋大事、谋发展，集中更多的时间抓项目、抓落实。今年省定十大实事，结合我市实际已量化分解到各项工作中，各级政府要以高度的责任感，办好这些惠民实事，改善民生，促进和谐。

　　（四）坚持从严治政，树立清廉形象。坚持标本兼治、综合治理、惩防并举、注重预防的方针，进一步完善惩治和预防腐败体系。紧紧围绕腐败易发多发的重点领域和关键环节，推进制度建设和改革创新，努力从制度上堵塞滋生腐败的漏洞，真正做到用制度管权、管事、管人。加强对权力的制约，特别要加强对领导干部、人财物管理使用、关键岗位的监督和制约，防止权力失控、决策失误、行为失范。坚持勤俭办一切事业，珍惜民资民力，严格控制新建楼堂馆所，坚决反对挥霍奢侈、铺张浪费。继续加强领导干部经济责任审计，加强对重大项目的审计和监督，扎实开展治理商业贿赂专项工作，对违法违纪行为严肃查处，决不姑息。全体政府工作人员特别是领导干部一定要勤政廉政、自警自励，做遵纪守法的模范，做廉洁奉公的表率，以实际行动取信于民，进一步营造风清气正的行政环境。

　　各位代表，加快发展是形势的需要、时代的强音；富民强市是人民的愿望、政府的职责。在困难和挑战面前，唯有实干才有出路，唯有发展才有希望。让我们在中共周口市委的坚强领导下，团结和带领全市人民，以更加昂扬的斗志、更加创新的精神、更加务实的作风，抢抓机遇，奋力追赶，勇往直前，攻坚克难，齐心协力，真抓实干，以优异成绩迎接新中国成立６０周年！
